# How Structure Shapes Dynamics: Knowledge Development in Wikipedia - A Network Multilevel Modeling Approach

**DOI:** 10.1371/journal.pone.0111958

**Published:** 2014-11-03

**Authors:** Iassen Halatchliyski, Ulrike Cress

**Affiliations:** 1 Knowledge Construction Lab, KMRC – Knowledge Media Research Center, Tuebingen, Germany; 2 Department of Applied Cognitive Psychology and Media Psychology, University of Tuebingen, Tuebingen, Germany; Semmelweis University, Hungary

## Abstract

Using a longitudinal network analysis approach, we investigate the structural development of the knowledge base of Wikipedia in order to explain the appearance of new knowledge. The data consists of the articles in two adjacent knowledge domains: psychology and education. We analyze the development of networks of knowledge consisting of interlinked articles at seven snapshots from 2006 to 2012 with an interval of one year between them. Longitudinal data on the topological position of each article in the networks is used to model the appearance of new knowledge over time. Thus, the structural dimension of knowledge is related to its dynamics. Using multilevel modeling as well as eigenvector and betweenness measures, we explain the significance of pivotal articles that are either central within one of the knowledge domains or boundary-crossing between the two domains at a given point in time for the future development of new knowledge in the knowledge base.

## Introduction

The social web affords natural interaction dynamics among a large number of participants. From the active participation of a multitude of users with different backgrounds and goals [1] emerge virtual communities [2] that define and follow their own overarching goals. The resulting process often takes the form of mass collaboration [3].

The interplay between the individual and the social in a self-organizing system of mass collaboration is based on the creation and use of shared digital artifacts that is enabled by Web 2.0 technologies (cf. [4], [5]). Individuals externalize their knowledge into artifacts [6], building a digital knowledge base with a network structure of interlinked contributions. This collective knowledge of a community (cf. [7]) is an emergent phenomenon [8] of amalgamation of diverse contributions in a discourse-like process through referring, modifying and building on each other. Each new contribution needs to be adequately integrated into the existing structure. As new knowledge in the form of concepts, connections and facts is introduced to the knowledge base, the collective knowledge of the community develops in a continuous process (cf. [9]).

The present paper reports on a research endeavor to model and test the significance of a generative mechanism for the development of collective knowledge in Wikipedia. We relate the dynamics of knowledge to its structural dimension. We thus provide an example of a methodological approach to research questions concerning the structure and dynamics of knowledge in mass collaboration contexts.

Wikipedia is a prominent Web 2.0 community with pronounced knowledge-related activities. It follows a “no original research” rule, meaning that it accommodates only previously published facts. However, those facts stemming from external sources are then integrated in an original way (cf. [10]). Thus, Wikipedia's knowledge base is a novel product of the community and undergoes development processes that are typical of genuine knowledge-building communities [6], [11]. In Wikipedia, knowledge develops on many levels of the created artifact: Article content is edited by adding, modifying or deleting parts of it and thus changing its textual structure; hyperlinks are extensively used to establish connections between articles; new articles are constantly created building up the knowledge domain as well as connecting different domains. System rules and community practices are the backbone for such developments and also experience changes over time themselves [12].

In the presented empirical study, we use a longitudinal network analysis approach to investigate the development of the knowledge base in Wikipedia by considering its structural properties. Focusing on two adjacent knowledge domains – psychology and education – and their intersection, we analyze the networks of knowledge consisting of interlinked articles and their development over 7 yearly snapshots from 2006 to 2012. Using multilevel modeling, we explain the significance of structurally pivotal articles located within or across the domains at a given point in time for the future appearance of new knowledge in the knowledge base.

## Measuring Development in Networks of Knowledge

Online mass collaboration promises high potentials for development of one of the most important factors in society nowadays – knowledge. Extensive research has been done on the conditions for attracting and maintaining a critical mass of participants in virtual communities that are motivated to contribute actively [13], [14], [15]. Little is known about the complex patterns of self-organization when new knowledge is developed within a community. Indeed, the prediction of radical innovations as a research goal is impossible by definition [16]. Based on a historical account of the developed knowledge, however, it is possible to notice promising directions for further advancement. As we intend to show in the present paper, it is also possible to model and measure relevant conditions and processes of knowledge development in a virtual community.

A suitable perspective on shared online knowledge is provided by the concept of a network. Big data sets of different collaborative networks such as interconnected scientific works, hyperlinked Wikipedia articles and many others are abundantly easily accessible on the Internet and have contributed to the rise of a “new science of networks” [17]. Webometric research, for example, adapts appropriate methods following a direct analogy between the analysis of scientific citations and of webpage hyperlinks as signs of knowledge relations or diffusion processes [18]. This analysis perspective suggests that the meaning of a single scientific paper or webpage in such networks is structurally defined by the presence and absence of relations to other works and by its specific position in the network as a whole [19]. Therefore, well-connected and central works in a network tend to contain pivotal knowledge for the collaborative community. These network nodes are marked by high interest or quality and have an impact as hubs or brokers of knowledge [20]. Among the various measures of network centrality, eigenvector [21] is a popular indicator for global hubs and betweenness [22] is a popular indicator for global brokers.

Network science has only lately started to expand its limited focus on static measures and structures in order to acknowledge the dynamics of complex networks. The simplest approach is a description of indicators changing over a specific time interval. Global and local network measures can be represented as a series of snapshots at different points in time. Temporal analyses of networks thus usually describe developments based on differences between snapshots [23]. This has also been done for the articles and authors in Wikipedia [24], [25], [26]. More complex approaches to network dynamics are necessary in order to explain the appearance of new knowledge based on change processes in existing knowledge or to explain the continuously changing position of existing ideas in a knowledge network in light of new emerging knowledge [27]. For the network of Wikipedia articles, such analysis could seek to establish a relation between the network position of existing interconnected articles, the change in their position over time, and the appearance of new knowledge in the form of new articles or new contributions to specific articles in the network.

The links of pivotal nodes in real-world networks are usually distributed according to a so-called power law, that is, there are very few hubs with a very high number of connections and a mass of network nodes with just a few connections. The more citations a paper has already received, the more new citations it is likely to receive. The “rich get richer” principle has been widely acknowledged in models of network growth as an explanation of such inequalities in the frequency distribution of pivotal, well-connected nodes in a network. For scientific networks, this principle was called “the Matthew effect” by [28] in reference to the Gospel of Matthew and also “cumulative advantage” by [29] later on. [30] finally coined the term “preferential attachment” and specified a network evolution model with a continuously rising number of nodes. According to this generative mechanism, new nodes are linked with a higher probability to well-connected than to poorly connected nodes among the already existing ones.

In networks of Wikipedia articles, pivotal nodes have been regarded in the context of adjacent knowledge domains and related to contributions by experienced authors in the community [31]. As the distribution of article hyperlinks follows a power law [26], the probability that an article will receive new links is proportional to its degree, that is, the number of its existing connections with other articles. Correspondingly, the preferential attachment mechanism has been verified for Wikipedia and also for the Web as a network of websites [32]. Assuming that the elaborate system of rules in Wikipedia is strictly followed (cf. [33]), the hyperlink structure of Wikipedia articles, which is also regulated extensively (see, for example, [34]), is a reliable representation of an extensive knowledge repository and reflects the internal structure of encyclopedic knowledge (cf. [35]). The preferential attachment rule could be extended to explain the process of knowledge development in Wikipedia. Thus, the appearance of new knowledge could be related to the existing structurally pivotal knowledge.

In the following, we present an empirical study with a longitudinal design that models the development of knowledge in the Wikipedia knowledge base. Employing a network analysis approach, we measure the topological position of articles within networks over a series of yearly snapshots in order to identify pivotal articles and their change over time. Our goal is to test the significance of structurally pivotal articles for the subsequent appearance of new knowledge in future periods and thus to capture the interplay between structure and dynamics of knowledge.

## Method

### Data

We investigated the relevant factors for development of new knowledge in Wikipedia, focusing on the two related knowledge domains *psychology* and *education*. Our data was sourced from an official dump file of the German Wikipedia [36], containing a snapshot of its state as of January 16, 2012.

All articles categorized as topics of psychology (German: “Psychologie”) or education (“Pädagogik”) as well as all their subcategories entered the study. The sample represented two knowledge domains with a similar number of articles and obvious content relations. Based on the content history of the past versions of these articles in the dump file, we took six successive snapshots of the two domains. Each of the snapshots referred to the same date, January 16, with an interval of one year between the snapshots. The first snapshot reflected the state of knowledge at the beginning of 2006, and the last (seventh) snapshot reflected the state of knowledge at the beginning of 2012. For the final dataset with all measures used in the study, we refer the reader to Text S1.

### Measures

We considered three types of articles in the analysis: specialized education articles, specialized psychology articles, and intersection articles (i.e., those categorized under both domains). Beginning by categorizing the final snapshot, which we regarded as the best developed, we traced back whether each categorized article existed in each preceding snapshot. The numbers of categorized articles over the years are shown in Table 1. For each article we recorded its year of creation and subtracted 2006 as a reference year from it (cf. [37]). We took into account which articles were distinguished by the German Wikipedia community as featured articles for their exceptionally well-written content. In order to differentiate the controversiality of the article topics, we used the algorithm developed by [38]. Explanatory variables that changed over the time span of the study were the total number of article edits at each snapshot year and the article age in years since creation. In order to make inferences about only the knowledge-related development of the articles, we first excluded from the analysis edits marked as minor, made by anonymous authors or bots, deletions, reversions to a previous article state of the article, as well as contributions shorter than 150 characters. We also excluded the contributions of administrators and reviewers. Although they contribute a large amount of content, their choice of articles and mode of contribution is driven by particular reasons reflecting their administrative responsibilities in Wikipedia. Moreover, [25] have shown that the percentage of contributions from such authors dramatically declined after 2004.

**Table 1:** Development of the number of categorized articles and of authors.

snapshot year	specialized psychology articles	specialized education articles	intersection articles	Σ articles	Σ authors
2006	2176	1357	325	3858	1776
2007	2911	1980	450	5341	3113
2008	3472	2556	526	6554	4265
2009	3908	3108	581	7597	5251
2010	4262	3595	626	8483	6104
2011	4660	4166	686	9512	7047
2012	5085	4696	731	10512	8002

We used network analysis in order to measure how pivotal each article was at a given point in time. Pivotal network position was regarded as an expression of an article's significance in the structural dimension of knowledge. For each of the seven snapshots of the knowledge domains, we extracted the current networks of knowledge at that time by parsing the relevant hyperlinks from the content of the last version of each article on each January 16. The networks of knowledge consisted of articles as nodes and of the hyperlinks between them transformed into undirected edges. Thus, the networks were aimed at representing the conceptual structure of knowledge in the interrelated articles and not the browsing and diffusion processes that only flow in the direction of the hyperlinks. For each snapshot, we constructed the two single domain networks, one for psychology and one for education, as well as the combined network of both domains. The extent to which an article was boundary-crossing between both domains was determined by measuring its betweenness [22] in the combined network at each of the seven points in time. Analogously, in each of the two separate domain networks we used eigenvector centrality [21] to measure how well-connected and thus central each article was. The pivotal articles in each of the snapshots were those with higher values of either betweenness or eigenvector centrality. The network analysis measures were calculated with the igraph package for R [39].

New knowledge in Wikipedia appears in the form of either newly created articles or new edits that add information to existing articles. As we wanted to locate the development of new knowledge in the network of articles, we operationalized the concept of new knowledge for each article in each period in three ways: first, by counting its new neighboring articles that were created in that period, that is, directly hyperlinked articles with an age of less than one year; second, by calculating the change in the sum of edits to all the neighboring articles in that period; and third, by counting the number of new contributions an article received in that period.

### Modeling approach

Our data consisted of article variables some of which were measured repeatedly and others that were time invariant characteristics. The longitudinal study design naturally lent itself to multilevel analysis, which is a state-of-the-art approach in educational research [40], [41]. The dependency of the repeated measures of the same articles was taken into account by differentiating two levels: the level of the measurement period and the level of the articles. Statistical calculations were executed with the *lme4* package for R [42].

## Hypotheses

The major goal of this longitudinal study was to explain the appearance of new knowledge in Wikipedia by focusing on the networks of hyperlinked articles within and across two domains. Our hypotheses therefore address the variables betweenness and eigenvector centrality, which measure how pivotal the position of an article is in a network for a given period snapshot. Deriving from the preferential attachment rule [30], [32] that an article receives new hyperlinks with a probability proportional to the number of its existing hyperlinks to other articles, we formulate the following hypotheses:

Hypothesis 1: Articles with a pivotal network position within or across knowledge domains become linked with a higher number of new neighboring articles during the subsequent period than do less pivotal articles.

Hypothesis 2: The neighbors of pivotal articles receive more new contributions than the neighbors of less pivotal articles during the subsequent period.

Hypothesis 3: Pivotal articles receive a higher number of contributions during the subsequent period than do less pivotal articles.

In order to more accurately evaluate the main effect in each of these hypotheses, it is simultaneously evaluated with the partial effects of a number of control variables: article type, year of creation, age, number of received contributions, number of neighbors, featured article distinction and article controversiality.

Each of the hypothesis tests were carried out using separate models for the psychology, the education and the combined networks.

## Results

### Descriptive trends

Before we present the statistical tests of the hypotheses, we first provide a descriptive overview of the development of the articles and authors in the domains between 2006 and 2012. This information outlines the state of Wikipedia before and during the longitudinal study interval and thus introduces to the context of our investigation. Table 1 shows a continuous increase in the number of articles and authors. A detailed investigation of the number of articles before the studied time interval revealed an increasing growth rate until the peak year 2005. The author growth rate in the studied domains rose until the peak year 2007, that is, for two years longer than that of the articles. In later periods, as depicted in Table 2, the number of both articles and authors had diminishing growth rates and reached a steady level of growth by 2008/2009.

**Table 2:** Yearly growth in the total number of articles and authors.

	2002/03	2003/04	2004/05	2005/06	2006/07	2007/08	2008/09	2009/10	2010/11	2011/12
articles	119	574	1658	1486	1483	1213	1043	886	1029	1000
authors	12	135	677	952	1337	1152	986	853	943	955

Considering the number of articles that received new contributions during a one-year period between two snapshots, Table 3 shows that it had been rising until 2007/2008 when it reached a stable level for specialized psychology articles and for intersection articles. The number of edited education articles per period rose throughout the studied interval, albeit slower since 2008.

**Table 3:** Development of the number of articles with new contributions per period.

	2006–2007	2007–2008	2008–2009	2009–2010	2010–2011	2011–2012
psychology	1285	1561	1541	1428	1451	1515
education	739	955	1026	1049	1071	1161
intersection	198	269	252	210	233	231
Σ	2222	2785	2819	2687	2755	2907

**Figure 1:**
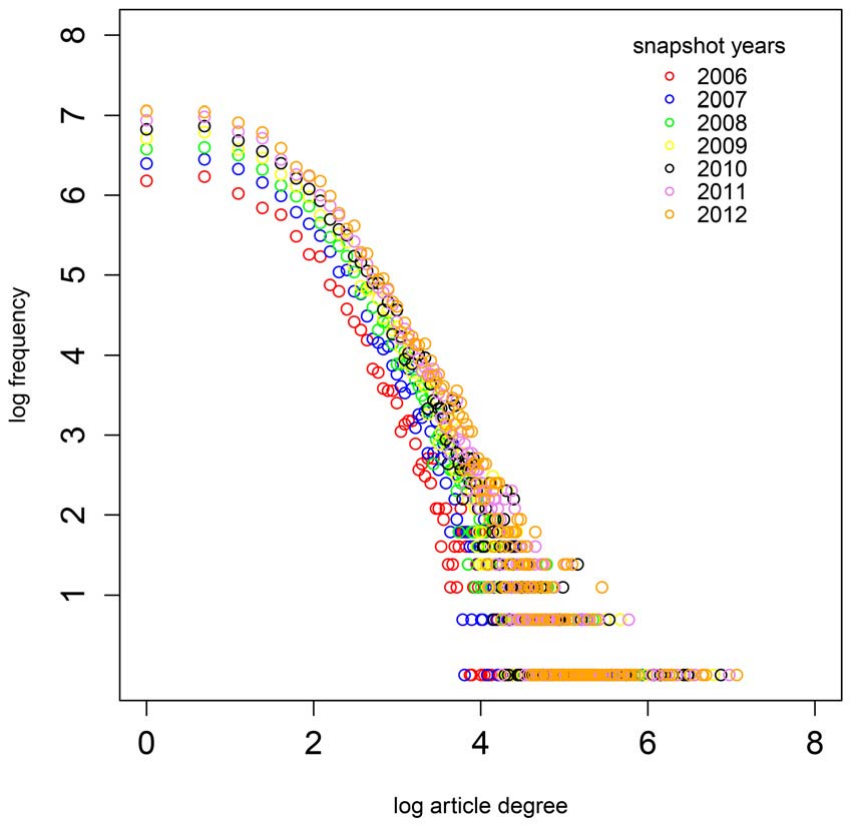
Degree distribution in the combined network of psychology and education articles in the seven snapshot years. In a log-log scale, the colored points display the frequency of articles with a given number of neighbors over the years.

Regarding the network of articles, we observed a stable power law degree distribution in all the snapshots as displayed in Figure 1. Due to the growth in the network the degree distribution shifts upwards over time. The degree distributions of the psychology, education and intersection articles in the single domain networks show the same pattern.

**Figure 2:**
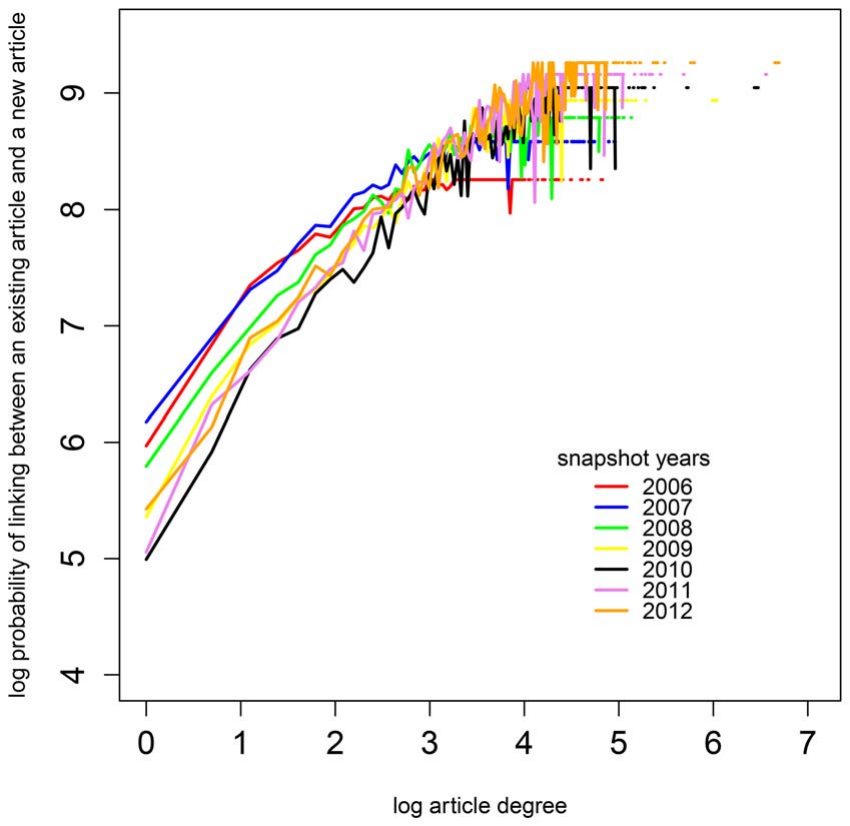
Preferential attachment in the combined network of psychology and education articles in the seven snapshot years. In a log-log scale, the colored lines demonstrate the relationship between the degree of an article and its probability to be linked to a newly created article for each of the yearly snapshots.

Our data partly confirmed the results of [32] who demonstrated preferential attachment for the main English and Portuguese Wikipedia networks with decreasing linking probability for the articles with very high degree, that is, number of neighbors. As Figure 2 shows, the preferential attachment for the combined network of psychology and education articles becomes saturated for large values of the degree of an article. Our data consists of discrete network snapshots in time, and we cannot observe well differences in the linking probability among articles with high degree that become linked to new articles in each period. In addition, Figure 2 reveals a decrease in the linking probability of low-degree articles and an increase in the linking probability of high-degree articles over the years.

The percentage distribution of links between the different types of articles in the network remained stable over the snapshots. The connections with articles outside the two domains of focus held the largest share in both domain networks: 91% in psychology and 84% in education. The connections between intersection articles and strictly psychology articles amounted to 8%, and between intersection articles and strictly education articles amounted to 15%. The connections between strictly psychology and education articles amounted to 1% in each of the domain networks.

In summary, the descriptive analysis revealed that the number of newly created articles during a one-year period was the first variable that stopped growing, with the growth rate diminishing around 2005. By 2009 the dynamics of articles and authors in the analyzed Wikipedia domains demonstrated stability. These results indicate that the studied interval was well-chosen and the hypotheses tests are not likely to be biased by exogenous disturbances of the dynamics of the mass collaboration system.

### Hypotheses testing

Our hypotheses concern the appearance of new knowledge in the article networks of two knowledge domains. According to Hypothesis 1, we first modeled the appearance of new knowledge as the number of newly created articles that become direct neighbors of an article in each of the three networks (i.e., psychology, education and combined) during each one-year period in the studied time interval.

The need for employing multilevel modeling was confirmed by the calculated design effects, which were all greater than 2 (cf. [43]): 2.65 for psychology, 2.47 for education and 2.43 for the combined network. The dependent variable, the number of newly created articles as neighbors, is a count variable with a high percentage of zeros throughout the measurement instances: 69.1% in psychology, 74.0% in education and 72.0% in the combined network. Therefore we used logistic models that treat the number of new articles as a binary outcome variable, that is, they model the differences between cases with zero versus cases with a non-zero count of new articles as neighbors. The general model specification was:
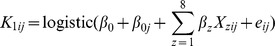
where 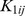 denotes as 1 or 0 whether article *i* has received at least one newly created article as a neighbor between the snapshot periods *j-1* and *j*, 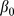 is the global fixed intercept, 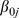 is the random intercept for each of the seven snapshots, 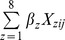 is the linear combination of the eight explanatory variables and their regression coefficients and 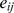 is an error term. Table 4 shows the results of the regressions successively for the psychology, education and the combined network. The column *level of variable* indicates whether the variable is time invariant for each article or it is repeatedly measured in each period.

**Table 4:** Multilevel logistic models of receiving newly created articles as neighbors.

	Level of variable	Estimate	Std. Error	z value	Pr(>|z|)
Combined					
(Intercept)		2.56	0.11	23.19	<2e-16***
creation year	article	−0.33	0.01	−25.27	<2e-16***
article age	period	−0.22	0.01	−21.57	<2e-16***
t-1 log betweenness	period	0.31	0.01	33.30	<2e-16***
t-1 log edit count	period	0.26	0.02	11.40	<2e-16***
education article	article	−0.17	0.04	−4.28	1.9e-05***
intersection article	article	0.19	0.07	2.87	0.0041**
featured article	article	0.04	0.19	0.20	0.8399
log controversiality	article	0.05	0.01	4.26	2.0e-05***
Psychology					
(Intercept)		1.55	0.10	15.33	<2e-16***
creation year	article	−0.47	0.02	−27.58	<2e-16***
article age	period	−0.33	0.01	−26.14	<2e-16***
t-1 log eigenvector	period	0.51	0.02	26.82	<2e-16***
t-1 log edit count	period	0.37	0.03	12.894	<2e-16***
intersection article	article	0.68	0.07	9.50	<2e-16***
featured article	article	−0.14	0.22	−0.61	0.5430
log controversiality	article	0.06	0.01	4.32	1.5e-05***
Education					
(Intercept)		−0.20	0.10	−1.89	0.0586.
creation year	article	−0.38	0.02	−19.97	<2e-16***
article age	period	−0.18	0.01	−12.47	<2e-16***
t-1 log eigenvector	period	0.27	0.02	15.69	<2e-16***
t-1 log edit count	period	0.47	0.03	13.80	<2e-16***
intersection article	article	0.70	0.08	9.04	<2e-16***
featured article	article	0.37	0.40	0.91	0.3605
log controversiality	article	0.08	0.02	4.15	3.3e-05***

The models for the three networks were congruent with each other. They all featured the same set of significant regressors. Regressors with a significant negative influence were article creation year and age, that is, the later the year of creation of an article in Wikipedia and the more years since its creation, the less likely it was that the article received any newly created articles as neighbors. In support of Hypothesis 1, both an article's previous period betweenness (t-1 log betweenness) in the combined network and an article's previous period eigenvector centrality (t-1 log eigenvector) in the psychology or education network were regressors with a significant positive influence, as was the number of contributions received up to the previous period in all three networks (t-1 log edit count). Intersection articles were significantly more likely than specialized psychology or education articles to receive newly created articles as neighbors. Featured articles were not significantly different from non-featured articles in their probability to receive newly created articles as neighbors. Article controversiality was a significant positive regressor.

To test Hypothesis 2, we next modeled the dynamics of new knowledge as the change in the total edit count of the neighboring articles of an article during one period. Again, multilevel modeling was necessary as the design effects were all greater than 2: 2.72 for psychology, 2.47 for education and 2.56 for the combined network. The distribution of the dependent variable permitted the use of linear multilevel models. The general model specification was:
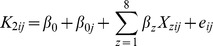
where 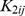 denotes as 1 or 0 whether article *i* has received at least one newly created article as a neighbor between the snapshot periods *j-1* and *j*, 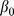 is the global fixed intercept, 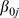 is the random intercept for each of the seven snapshots, 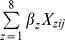 is the linear combination of the eight explanatory variables and their regression coefficients and 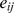 is an error term. The results are presented in Table 5 successively for the psychology, education and the combined network.

**Table 5:** Multilevel linear models of the change in the edit count of the neighboring articles.

	Level of variable	Estimate	Std. Error	t value	Pr(>|z|)
Combined					
(Intercept)		131.95	4.09	32.29	<2e-16***
creation year	article	−7.73	0.48	−15.97	<2e-16***
article age	period	−8.13	0.35	−23.32	<2e-16***
Δ neighbors since t-1	period	20.42	0.11	180.30	<2e-16***
t-1 log betweenness	period	5.86	0.33	17.82	<2e-16***
t-1 log edit count	period	9.98	0.90	11.82	<2e-16***
education article	article	−37.04	1.67	−22.19	<2e-16***
intersection article	article	−9.28	2.91	−3.19	0.0014**
featured article	article	53.85	8.55	6.30	3.0e-10***
log controversiality	article	6.28	0.50	12.68	<2e-16***
Psychology					
(Intercept)		116.88	3.47	33.66	<2e-16***
creation year	article	−9.02	0.57	−15.79	<2e-16***
article age	period	−9.06	0.43	−21.28	<2e-16***
Δ neighbors since t-1	period	23.66	0.14	171.27	<2e-16***
t-1 log eigenvector	period	13.30	0.58	22.80	<2e-16***
t-1 log edit count	period	13.19	1.04	12.74	<2e-16***
intersection article	article	0.79	2.73	0.29	0.7732
featured article	article	58.11	9.01	6.45	1.1e-10***
log controversiality	article	6.61	0.55	11.92	<2e-16***
Education					
(Intercept)		53.14	2.43	21.90	<2e-16***
creation year	article	−6.38	0.43	−14.79	<2e-16***
article age	period	−5.85	0.33	−17.66	<2e-16***
Δ neighbors since t-1	period	14.87	0.13	112.62	<2e-16***
t-1 log eigenvector	period	4.52	0.36	12.70	<2e-16***
t-1 log edit count	period	10.54	0.83	12.63	<2e-16***
intersection article	article	36.77	2.09	17.56	<2e-16***
featured article	article	1.10	11.27	0.10	0.9225
log controversiality	article	4.04	0.56	7.23	4.9e-13***

Generally, the results correspond with those from the previous models of the number of new articles as neighbors. The models for the three networks again featured nearly the same set of significant regressors. Regressors with a significant negative influence were again article creation year and age. The change in the number of neighbors since the previous period functioned as a control variable and had a high positive t-value in explaining the variance of the dependent variable, that is, the change in the total edit count of the neighbors. In support of Hypothesis 2, further regressors with a significant positive explanatory power were again the article's previous period betweenness in the combined network, the article's previous period eigenvector centrality in the psychology or education network, and the number of contributions received up to the previous period. Except in the psychology network, psychology articles had higher positive values of change in the edit count of the neighboring articles than intersection articles and intersection articles had higher positive values than education articles. Except in the education network, featured articles had significantly higher positive values of change in the edit count of the neighboring articles than non-featured articles. Article controversiality was again a significant positive regressor in these models.

Our third and last indicator of new knowledge was the number of new contributions an article receives during a one-year period (Hypothesis 3). Again, the calculated design effects required multilevel modeling: 2.39 for psychology, 2.16 for education and 2.27 for the combined network. In more than half of the data snapshots, the articles did not receive any new edits in the past year, so the dependent variable again contained an excess of zeros: 58.2% in psychology, 62.8% in education and 60.6% in the combined network. Using logistic models, we investigated the binary outcome, that is, the differences between occasions when articles received zero versus at least one new edit during a given period. The general model specification was:
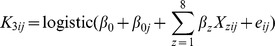
where 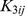 denotes as 1 or 0 whether article *i* has received at least one newly created article as a neighbor between the snapshot periods *j-1* and *j*, 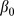 is the global fixed intercept, 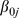 is the random intercept for each of the seven snapshots, 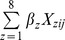 is the linear combination of the eight explanatory variables and their regression coefficients and 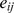 is an error term. Table 6 shows the results of the regressions successively for the psychology, education and the combined network.

**Table 6:** Multilevel logistic models of an article receiving new edits.

	Level of variable	Estimate	Std. Error	z value	Pr(>|z|)
Combined					
(Intercept)		0.63	0.09	7.11	1.2e-12***
creation year	article	−0.39	0.01	−34.40	<2e-16***
article age	period	−0.31	0.01	−32.86	<2e-16***
t-1 log betweenness	period	0.09	0.01	12.10	<2e-16***
t-1 log edit count	period	0.65	0.02	33.02	<2e-16***
education article	article	0.01	0.03	0.20	0.8420
intersection article	article	0.01	0.06	0.34	0.7350
featured article	article	0.18	0.19	0.94	0.35
log controversiality	article	0.20	0.01	16.34	<2e-16***
Psychology					
(Intercept)		−0.02	0.07	−0.21	0.8303
creation year	article	−0.43	0.01	−32.38	<2e-16***
article age	period	−0.33	0.01	−30.33	<2e-16***
t-1 log eigenvector	period	0.05	0.01	4.43	9.6e-06***
t-1 log edit count	period	0.68	0.02	29.94	<2e-16***
intersection article	article	0.11	0.05	1.98	0.0475*
featured article	article	0.17	0.21	0.83	0.4093
log controversiality	article	0.19	0.01	14.08	<2e-16***
Education					
(Intercept)		−0.25	0.08	−3.31	0.0009***
creation year	article	−0.37	0.01	−26.28	<2e-16***
article age	period	−0.31	0.01	−24.51	<2e-16***
t-1 log eigenvector	period	0.03	0.01	2.92	0.0035**
t-1 log edit count	period	0.72	0.03	27.67	<2e-16***
intersection article	article	0.10	0.06	1.83	0.0678.
featured article	article	0.42	0.34	1.23	0.2160
log controversiality	article	0.22	0.02	11.34	<2e-16***

The results of this third perspective on new knowledge in the networks followed the pattern of the previous two. In all networks, article creation year and age were regressors with a significant negative influence on the dependent variable. Significant positive regressors were the previous period betweenness and eigenvector centrality in support of Hypothesis 3, as well as the number of received contributions up to the previous period. Article type was only marginally significant, with intersection articles being more likely than specialized psychology or education articles in both separate domain networks to receive new contributions. Featured articles were not more likely to receive new contributions than non-featured articles. Finally, article controversiality had a significant positive explanatory power for our third indicator of new knowledge.

## Discussion

The aim of this longitudinal empirical study was to explain the appearance of new knowledge in Wikipedia by taking the article network structure into account and by focusing on the pivotal articles in particular. Articles with a pivotal network position within or across two domains at a given point in time were expected to be a connecting factor for larger amounts of new emerging knowledge during the following year than less pivotal articles. The expectation was operationalized for three forms of new contributed knowledge in Wikipedia: the number of new articles as neighbors, the change in the total sum of edits of the neighbors and the number of new received contributions.

The hypotheses of the study were supported by the empirical results. The tests showed that pivotal articles, indicated by high betweenness in the combined network or by high eigenvector centrality in the separate domain networks, link to all three relevant forms of new emerging knowledge in Wikipedia at a significantly high rate. In spite of the differences in the network positions of these articles within and across the knowledge domains, both types of articles are pivotal for knowledge development.

According to our results shown in Figure 2, the probability of an article to receive newly created articles as neighbors increases with its degree. Thus, the results of testing Hypothesis 1 shown in Table 4 can also be interpreted as evidence of the relationship between the centrality indicators degree and betweenness, and between degree and eigenvector. Indeed, these are distinct indicators, all of which point out the relative significance of nodes in a network. A node with high degree also has a high probability to be part of the shortest connection between many of the other nodes. Thus, degree is related with betweenness, at least in non-fractal networks [44], [45]. Eigenvector is also related with degree as it extends the count of neighbors of a node by taking the degree of these neighbors into account [46].

The explanatory power of pivotal articles for new emerging knowledge has been substantiated through the inclusion of a number of significant control variables in our models. These variables expand the preferential attachment mechanism [30], which we have shown for the knowledge development in Wikipedia, by a number of other important effects. The model results showed that the later the starting year of an article and the older its age, the less likely it links to new emerging knowledge. Empirical evidence from scientometric studies also indicated the effect of aging of scientific work beyond the preferential attachment mechanism as the age of a scientific work is negatively related to its likelihood of receiving future citations, and thus to its impact on future knowledge development [47]. It seems that pivotal articles in Wikipedia were created in the early years of the studied domains. At that time, there was more intensive work on creating new articles and developing pivotal articles. The positive effect of the network position of pivotal articles on knowledge development is strong enough to supersede the negative effect of their higher age.

Our results furthermore showed that articles with many contributions and intersection articles of two domains are largely more likely to link to new emerging knowledge. Independent of whether they are pivotal in the network, articles on topics that receive much attention have the potential to generate further knowledge development, at least for a short time. This supports the results of [48], who showed that popular and relevant article topics receive a high number of contributions and are likely to be of high quality. Featured article distinction can be regarded as an additional factor of knowledge development that only concerns the neighborhood of a featured article. Article controversiality was demonstrated to increase all three types of knowledge development.

It is important to note that our results about pivotal and new knowledge apply to specific knowledge domains and to a specific stage in the historical development of the German Wikipedia. Following our descriptive analysis of the studied time interval, the rates of growth in the number of articles, authors and contributions largely reached stable levels after a short interval of decrease. We would regard the preceding interval until 2006 as a different stage in the history of the German Wikipedia, as it grew exponentially [49]. observed the same pattern in the English Wikipedia. As already mentioned, before the articles' growth rate began to diminish, it peaked in 2005 and thus 2 years earlier than the peak of the authors' growth rate. We see this as evidence that a new stage of the German Wikipedia's history, which was framed by the present study, was initiated by the stagnating number of new articles. We call this stage of stable growth rates an equilibrium stage. Studying the development of scientific fields [50], regularly observed a similar saturation stage after knowledge had grown exponentially, and after opportunities for incremental developments had finished. As Wikipedia is an evolving complex system, it is unclear how stable its equilibrium might be and what internal or external processes might currently protect or endanger it.

In a study that first recognized this stagnation [49], noted three possible causes for it at the level of authors: conflicts between experienced and new authors; bureaucracy with rising coordination costs for the contributors; and deficient collaborative tools. For the level of articles [49], also conjectured that the number of available new encyclopedic topics that still had not been covered in Wikipedia might be declining [51]. later doubted the relevance of this knowledge saturation hypothesis and pointed out that even if this were the case, there would still be a plenty of writing that could be done, as even some of the most important Wikipedia articles would suffer from bad quality. While our study did not directly test the hypothesis of whether worsened conditions of collaboration had slowed down the German Wikipedia's growth, our results indicate that this probably came as a later factor in a longer causal chain. Its origin seems to have been the reduced choice of new articles on accessible, well-known topics that could still be created.

The creation of new articles did not come to a halt but went back to a lower linear yearly rate. In the new equilibrium stage, articles in the studied domains of the German Wikipedia presumably required more specialized knowledge and greater cognitive efforts than in the earlier exponential growth stage. Table 3 also showed that the number of articles with new contributions per period continued growing without a decline and then became stable. A plausible reason for this is that some of the efforts for creating and expanding new articles were switched to other older articles.

The declining availability of popular topics that have not yet been written affected the numbers of new, inexperienced authors. As we have shown in our previous investigations [52], [31], author's experience in contributing to different articles is needed in order to be able to contribute to pivotal articles that have reached advanced stages of development and make up the structure of a knowledge domain.

## Conclusions

The interplay between structure and dynamics in knowledge-related networks has been pointed out as a promising area of research [53]. The present work applied powerful, longitudinal multilevel analysis and showed that structures that are pivotal within the static organization of knowledge are also pivotal for the dynamic development of new knowledge measured in three ways in Wikipedia. Thus, the results integrate with our previous investigations [52], [31] of the contribution experience of authors that substantially promote the appearance of new knowledge by contributing to pivotal articles.

The presented results, however, also raise some critical thoughts about the mass collaboration system Wikipedia. Associated with the reduced availability of new topics, the online encyclopedia as a whole has reached a saturation stage after an initial exponential growth. Participation thresholds facing relatively inexperienced authors are continuously rising, and the work that remains can only be performed by a tiny percentage of authors who have acquired authority status [54].

Although we cannot be sure about the transferability of the insights gained from Wikipedia, we found evidence that the structures and dynamics of knowledge development exhibit mechanisms similar to other knowledge-related realms like scientific work. This encourages us to look further into generally relevant conditions and processes and to embrace the challenge of the dynamics of knowledge, which is difficult to grasp (see, for example, [55], [56]). Series of ideas and actions are said to lead to the stabilization of historical trajectories and structural patterns over time [27]. We find it interesting and important to deepen our understanding of the dynamics of this major factor in society – knowledge.

## Supporting Information

Text S1 **Dataset with all measures used in the study.**
(ZIP)
